# Electrocardiographic Abnormalities in *Trypanosoma cruzi* Seropositive and Seronegative Former Blood Donors

**DOI:** 10.1371/journal.pntd.0002078

**Published:** 2013-02-28

**Authors:** Antonio L. Ribeiro, Ester C. Sabino, Milena S. Marcolino, Vera M. C. Salemi, Barbara M. Ianni, Fábio Fernandes, Luciano Nastari, André Antunes, Márcia Menezes, Cláudia Di Lorenzo Oliveira, Vandana Sachdev, Danielle M. Carrick, Michael P. Busch, Eduard L. Murphy, for the NHLBI Retrovirus Epidemiology Donor Study-II (REDS-II), International Component

**Affiliations:** 1 Hospital das Clínicas and Faculdade de Medicina, Universidade Federal de Minas Gerais, Belo Horizonte, Brazil; 2 Department of Infectious Disease and Institute of Tropical Medicine, University of São Paulo, São Paulo, Brazil; 3 Cardiomyopathy Unit ofthe Heart Institute (InCor) da Faculdade de Medicina da Universidade de São Paulo, São Paulo, Brazil; 4 Centro de Ciências Biológicas e da Saúde, Universidade Estadual de Montes Claros, Montes Claros, Brazil; 5 Campus Dona Lindu da Universidade Federal de São João del Rei, Divinópolis, Brazil; 6 National Heart, Lung and Blood Institute, Bethesda, Maryland, United States of America; 7 Westat, Rockville, Maryland, United States of America; 8 Blood Systems Research Institute (BSRI) and University of California at San Francisco, San Francisco, California, United States of America; US Food and Drug Administration, United States of America

## Abstract

**Background:**

Blood donor screening leads to large numbers of new diagnoses of *Trypanosoma cruzi* infection, with most donors in the asymptomatic chronic indeterminate form. Information on electrocardiogram (ECG) findings in infected blood donors is lacking and may help in counseling and recognizing those with more severe disease.

**Objectives:**

To assess the frequency of ECG abnormalities in *T.cruzi* seropositive relative to seronegative blood donors, and to recognize ECG abnormalities associated with left ventricular dysfunction.

**Methods:**

The study retrospectively enrolled 499 seropositive blood donors in São Paulo and Montes Claros, Brazil, and 483 seronegative control donors matched by site, gender, age, and year of blood donation. All subjects underwent a health clinical evaluation, ECG, and echocardiogram (Echo). ECG and Echo were reviewed blindly by centralized reading centers. Left ventricular (LV) dysfunction was defined as LV ejection fraction (EF)<0.50%.

**Results:**

Right bundle branch block and left anterior fascicular block, isolated or in association, were more frequently found in seropositive cases (p<0.0001). Both QRS and QTc duration were associated with LVEF values (correlation coefficients −0.159,p<0.0003, and −0.142,p = 0.002) and showed a moderate accuracy in the detection of reduced LVEF (area under the ROC curve: 0.778 and 0.790, both p<0.0001). Several ECG abnormalities were more commonly found in seropositive donors with depressed LVEF, including rhythm disorders (frequent supraventricular ectopic beats, atrial fibrillation or flutter and pacemaker), intraventricular blocks (right bundle branch block and left anterior fascicular block) and ischemic abnormalities (possible old myocardial infarction and major and minor ST abnormalities). ECG was sensitive (92%) for recognition of seropositive donors with depressed LVEF and had a high negative predictive value (99%) for ruling out LV dysfunction.

**Conclusions:**

ECG abnormalities are more frequent in seropositive than in seronegative blood donors. Several ECG abnormalities may help the recognition of seropositive cases with reduced LVEF who warrant careful follow-up and treatment.

## Author Summary

Chagas disease (ChD), caused by the protozoa *Trypanosoma cruzi*, is endemic in most Latin America countries and may be transmitted via blood transfusions. Cardiac disease is a major feature of chronically infected patients and may be lethal. Universal blood bank screening for ChD has been established in most Latin American countries, as well as in non-endemic countries with large immigrant populations, including the United States, Canada, Spain and Portugal. Blood donor screening leads to large numbers of new diagnoses of chronic *T. cruzi* infection. Counseling these individuals should address the recognition of those with more severe disease that deserve to be rigorously evaluated by experienced cardiologists and treated more promptly. The electrocardiogram is an important exam that can help in the recognition of cardiac disease and the evaluation of prognosis in ChD patients, but its role in blood donors has not been studied. The authors describe some electrocardiographic abnormalities that are typical of the infected blood donors, as well ECG abnormalities that help in the identification of those with severe cardiac involvement. These results may guide the evaluations of patients with incidentally detected *T. cruzi* infection from blood bank testing or public health screening.

## Introduction

Chagas disease (ChD), caused by a flagellate protozoon, *Trypanosoma cruzi (T. cruzi)*, is a major health problem in Latin America, where more than 8 million persons are infected [1], [2]. Chronic cardiopathy is the most important and severe manifestation of human Chagas disease, eventually affecting approximately 20% to 40% of those in the chronic phase of the disease [1], [2]. Due to migratory movements, ChD is now a world-wide challenge, since hundreds of thousands of chronically infected persons are now living not only in *T.cruzi* endemic countries but also in developed countries, mainly in Europe and the United States and Canada [3], [4]. Since one of the mechanisms of transmission of the disease is via blood transfusions, universal blood bank screening for ChD has been established in most endemic countries, as part of South American regional initiatives of elimination of transmission of the disease [5], [6]. Non-endemic countries with large immigrant populations including the United States, Canada, Spain and Portugal, have also begun to institute interventions to prevent blood-borne *T. cruzi* transmission [7]. Blood donor antibody screening results in large numbers of new diagnoses of chronic *T. cruzi* infection, most of them in the asymptomatic, indeterminate form of infection [8], [9]. Counseling these individuals should address the recognition of those with more severe disease that deserve to be rigorously evaluated by experienced cardiologists and treated more promptly.

Electrocardiogram (ECG), one of the most important tests in evaluation of ChD, is used to define the clinical stage of the disease with potential prognostic implications [10]. Most ECG studies of newly diagnosed ChD patients were performed decades ago, generally in patients identified by community or hospital based sampling; information on ECG findings in seropositive blood donors is lacking as is data relative to matched seronegative controls evaluated in parallel [11]–[17]. Additionally, most studies of ECG findings are not accompanied by systematic results that use core-lab reading of Echo and ECG results and codification by internationally accepted criteria, as the Minnesota Code for ECG findings [18].

As part of the National Heart, Lung and Blood Institute (NHLBI) Retrovirus Epidemiological Donor Study-II (REDS-II), we developed a study to evaluate the prevalence of ECG abnormalities in seropositive blood donors and to recognize typical ECG abnormalities associated with left ventricular dysfunction, the most important prognostic marker in ChD.

## Methods

### Overall study design

This study was conducted from July 2008 to October 2010, part of a retrospective cohort study in which *T. cruzi* seropositive blood donors identified by blood bank screening and well-matched seronegative donors. Using blood donation records from 1998–2002, we enrolled 500 *T.cruzi* seropositive subjects (250 from the city of São Paulo and 250 from the city of Montes Claros in the State of Minas Gerais) and 500 seronegative donors from the same time period, as previously described [19]. Recruited individuals underwent a health questionnaire, a medical evaluation, fasting blood sample collection for lipid profile, glucose analysis and NT-pro brain natriuretic peptide (NT-proBNP), an ECG and an Echo. Results of ECG and Echo were reviewed blindly by centralized reading centers. *T cruzi* antibody status was confirmed on plasma samples collected at the time of enrollment.

### Ethics statement

The study follows the Declaration of Helsinki of the Ethical Principles for Medical Research Involving Human Subjects, was approved by the Brazilian National Ethical Committee (CONEP # 1312/2006) and all subjects gave written informed consent.

### Blood bank screening and enrollment procedures

In 1998–2002, Fundação Pro-Sangue in São Paulo performed *T. cruzi* antibody screening using three serological methods: ELISA, hemaglutination and immunofluorescence for Chagas [20]. For the purpose of this study, we included as seropositive cases donors who were positive by all three assays at the time of donation and on a separate sample obtained at the time of counseling, generally one to four months after the donation. In Montes Claros, Hemominas screened all donations with two serological assays: ELISA and hemaglutination. All donors reactive to both assays at the time of donation and counseling were considered eligible for this study. Follow-up samples from all enrolled subjects were retested for T. cruzi antibody under code at the REDS-II Central Laboratory in the US using an FDA-approved assay manufactured by Ortho Diagnostics [21]. All 483 control donors tested negative on their follow-up samples collected at the time of enrollment and clinical assessments for this study. Of the 499 case donors who originally screened as seropositive at the time donation in 1998–2002, serum collected in 2008–2010 from 498 tested repeat reactive, while the samples from one donor tested just below the assay cutoff consistent with slowly progressive seroreversion [21].

### Measurements

A face to face *T. cruzi* risk factor and health history questionnaire was administered by a trained nurse in each site. The questionnaire collected detailed information regarding demographics, cities of residence, physical activity, medical history, exposure to *T. cruzi*, previous ChD diagnoses, cardiac and GI symptoms, past medical history and medication history. All subjects received a physical examination by a non-blinded physician with recording of height, weight, blood pressure, heart rate and physical exam findings.

Resting 12-lead ECG were recorded using the same model of machine at both sites (General Electric MAC 1200 electrocardiograph; GE Healthcare, Waukesha, WI) using standardized procedures. All ECGs were processed blindly by the central ECG laboratory (Epidemiological Cardiology Research Center, Wake Forest University, Winston-Salem, NC), where they were visually inspected for technical errors and inadequate quality and processed with the 2001 version of the GE Marquette 12-SL program. ECGs were analyzed electronically, with manual over-reading by trained cardiologists to ensure quality control. ECGs were classified by Minnesota code criteria [18] using variables that were derived from the median complex of the Marquette measurement matrix. In this study, major and minor ECG abnormalities were defined as previously established [22], modified to include ECG abnormalities typical of Chagas cardiomyopathy with prognostic significance, as frequent supraventricular or ventricular premature beats [10]. Old myocardial infarction (MI) on ECG was defined by the presence of major Q wave abnormalities (MC 1.1.x or 1.2.x) or minor Q waves abnormalities with ST segment or T-wave abnormalities (1.3.x and [4.1.x, 4.2, 5.1, or 5.2]) [18].

Echocardiographic studies were performed using Sequoia 512 ultrasound machine (Acuson, Mountain View, CA, USA) at São Paulo site and GE Vivid3 (GE Healthcare, Waukesha, WI) at Montes Claros site. Cardiac measurements were performed according to the guidelines of the American Society of Echocardiography [23]. Studies were recorded in digital format and all measurements were performed on digital loops using a Digisonics offline analysis station (version 3.2 software, Digisonics, Houston, Tex) at the Cardiovascular Branch, Echocardiography Laboratory, National Heart, Lung, and Blood Institute, Bethesda, Maryland. LV ejection fraction (LVEF) was calculated based on modified form of Simpson's biplane method [23] and, if atrial fibrillation is present, LVEF is estimated by a visual method. For this study, LV systolic dysfunction was defined as LVEF<0.50.

### Statistical analysis

Statistical analyses were conducted using SAS 9.2 and SPSS 18. Distributions of data were examined for normality by using Kolmogorov-Smirnov tests. Continuous variables were expressed as median [interquartile range (IQR)] and differences between seropositive and seronegative donors were compared using Wilcoxon-Mann-Whitney test, since these variables were not normally distributed. Categorical variables were summarized as counts and percentages and differences were compared using the Chi-square test or Fisher exact test. Association between quantitative ECG variables and LVEF was evaluated by Spearman correlation coefficient (rs). A p-value<0.05 was considered significant.

Receiver Operator Characteristic (ROC) curves were plotted in order to evaluate the accuracy of ECG measurements in detecting reduced LVEF and the area under the curve (AUC) was calculated. Sensibility, specificity and positive and negative predictive values of abnormal ECG, wide QRS duration (≥120 ms) and long QTc interval (>440 ms) were calculated with 95% confidence intervals. These cut points were selected since they are well-established in cardiology practice.

## Results

### ECG abnormalities in *T. cruzi* seropositive and seronegative blood donors

The study sample consisted of 499 seropositive and 488 seronegative donors. Seropositive had a higher proportion of non-white skin color and lower weight, body mass index, total cholesterol levels and pulse heart rate, as well as higher NT-proBNP values (table 1). LV systolic dysfunction was more commonly found in seropositive cases. The groups were comparable with regard to age, gender or other major cardiovascular risk factors (table 1).

**Table 1:** Demographic and medical characteristics of the study population, by *T.cruzi* antibody status.

	Seropositive	Seronegative	p-value*
	N = 499	N = 488	
**Site**			
**Sao Paulo**	255 (51)	239 (49)	
**Montes Claros**	244 (49)	249 (51)	
**Male gender**	261 (52)	241 (49)	
**Age (years)**	48 (40–57)	49 (42–58)	
**Skin Color**			0.0005
**White**	155 (31)	203 (42)	
**Black**	56 (11)	28 (6)	
**Mixed**	274 (55)	249 (51)	
**Other**	11 (2)	7 (1)	
**Refuse to answer/missing**	3 (1)	1 (<1)	
**Height (cm)**	163 (157–170)	165 (158–172)	
**Weight (Kg)**	71 (64–79)	73,5 (66–84)	<0.0001
**BMI**	26 (24–29)	27 (25–30)	0.0009
**Pulse (bpm)**	65 (60–72)	70 (60–75)	0.002
**Systolic BP (mm Hg)**	125 (114–140)	125 (115–140)	
**Hypertension (self-reported)**	113 (23)	119 (24)	
**Diabetes (self-reported)**	27 (5)	24 (5)	
**Total Cholesterol (mg/dL)**	195 (168–226)	204,5 (175–230)	0.025
**Smoking (current or former smoker)**	216 (43)	233 (48)	
**Glucose (mg/dL)**	87 (79–96)	87 (80–98)	
**LVEF<0.50** **	36 (7)	11 (2)	0.0002
**NT-pro BNP (pg/mL)**	48 (27–90)	37 (23–64)	<0.0001

Data is presented as median (interquartile range) or numbers (%).

* Reported if <0.05.

** Available in 497 seropositive donors and 461 seronegative donors. NT-proBNP: NT-pro brain natriuretic peptide.

Quantitative variables are shown in table 2. All ECG intervals were longer in seropositive donors, and both heart rate and HRV indexes had lower values. ECG abnormalities were similar in seropositive and seronegative donors, except for a higher frequency in seropositive subjects of right bundle branch block and left anterior fascicular block, isolated (16% vs. 2%, p<0.001 for RBBB and 15% vs. 2%, p<0.001 for LAFB) or in association (4% vs. 0, p<0.001); and a higher frequency of left ventricular hypertrophy in seronegative subjects (<1% vs. 1%, p = 0.004).

**Table 2:** Quantitative ECG measurements in *T.cruzi* seropositive and seronegative subjects.

ECG variable	Seropositive	Seronegative	p- value*
	N = 499	N = 488	
**Heart Rate (bpm)**	62 (57–69)	64 (58–72)	0.003
**PR duration (ms)**	160 (144–178)	156 (142–168)	0.007
**QRS duration (ms)**	90 (84–104)	88 (82–95,75)	<0.001
**QT duration (ms)**	420 (402–439)	412 (392–432)	<0.001
**QTc duration (ms)**	429 (412–447)	427 (411–442)	0.061
**HRV sdnn (ms)** **	16 (10–25)	22 (14–35)	<0.001
**HRV rmssd (ms)** **	18 (11–29)	24 (15–39)	<0.001

Data is presented as median (interquartile range).

* Reported if <0.05.

** HRV data available in 467 seropositive and 458 seronegative subjects. QTc: corrected QT interval by Bazett's formula; HRV: heart rate variability; rmssd: root mean square of successive differences in normal RR intervals; sdnn: standard deviation of all normal RR intervals.

Seropositive cases showed more abnormal ECGs (51% vs. 32%, p<0.001) than seronegative donors due to a higher prevalence of major (26% vs. 9%) ECG abnormalities. They also presented a higher number of major ECG abnormalities per tracing when compared to seronegative donors: 20% vs. 7% had one and 6% vs. 2% had 2 or more ECG abnormalities, respectively (p<0.001).

### ECG abnormalities in *T. cruzi* seropositive blood donors with and without LV dysfunction

**Figure 1:**
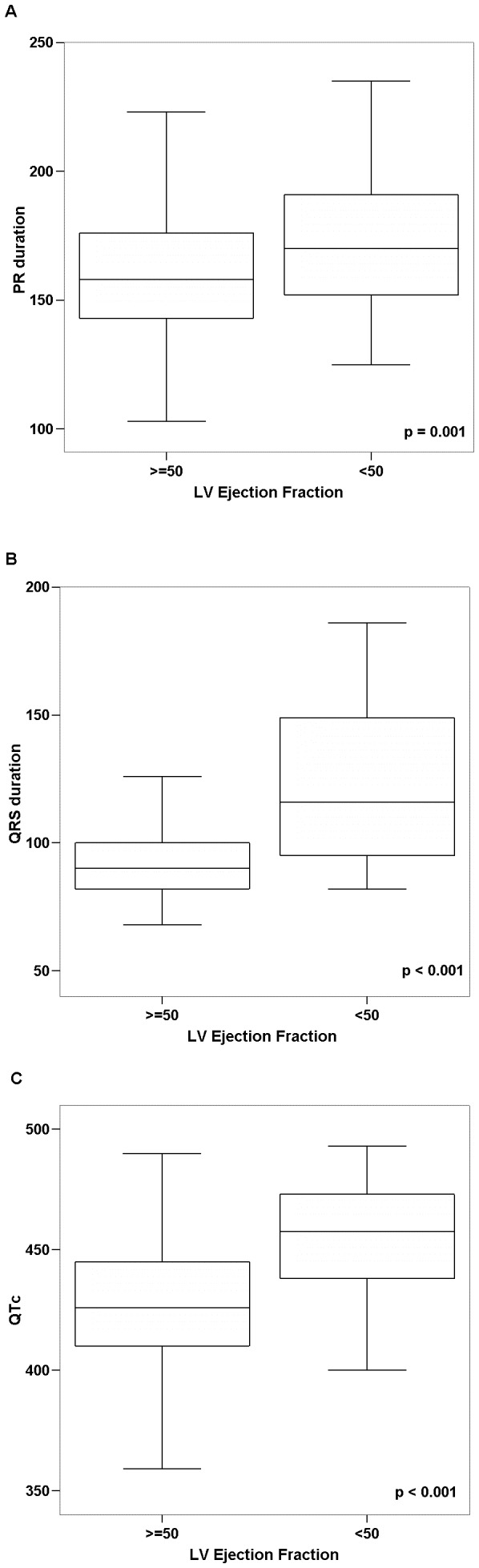
Quantitative ECG measurements in *T.cruzi* seropositive blood donors by left ventricular ejection fraction. A. PR interval. B. QRS duration. C. QT interval corrected by Bazett's formula.

**Figure 2:**
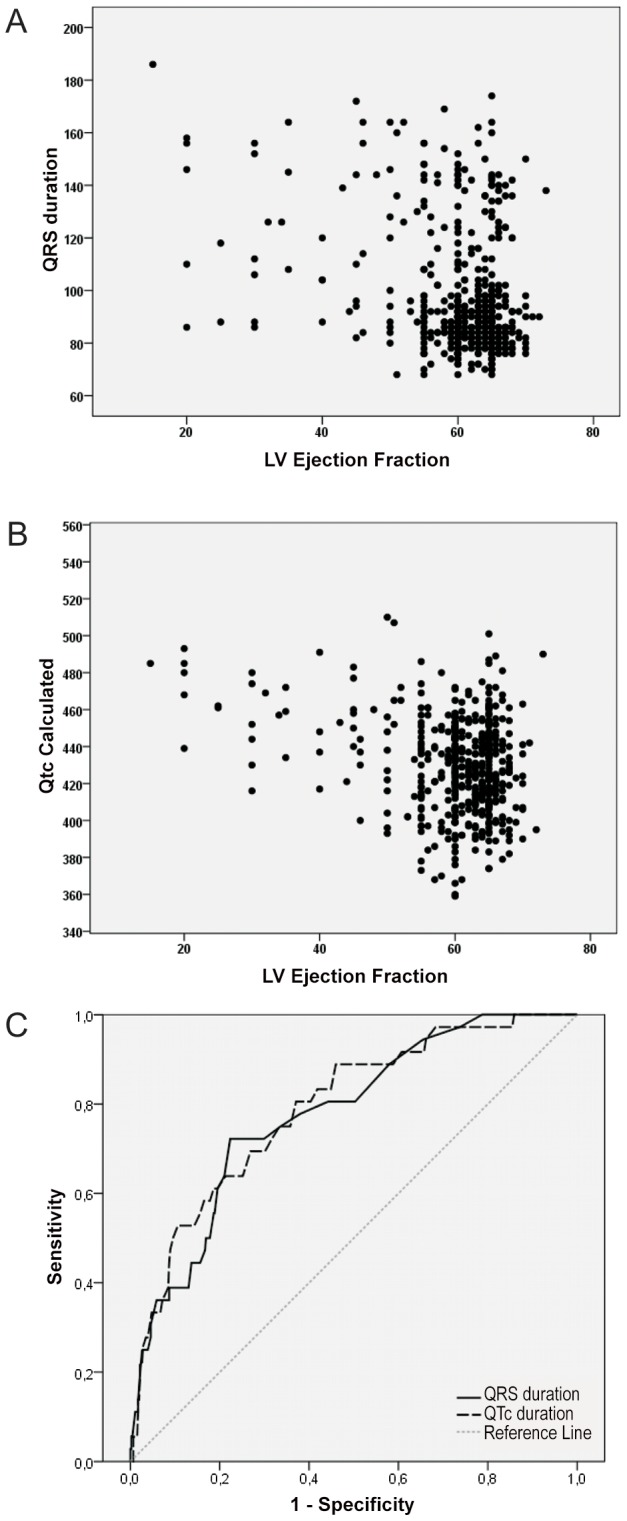
Association between by left ventricular ejection fraction and ECG measurements. A – Left ventricular ejection fraction and QRS duration; B - Left ventricular ejection franction and QT corrected interval; and C: Diagnostic accuracy (area under the ROC curve) of QRS duration and QT corrected interval in the detection of reduced left ventricular ejection fraction.

LVEF was reduced in 36 out of 497 seropositive subjects with available data (prevalence of LV systolic dysfunction of 7.2%); in two patients LVEF measurement was not obtained due to technical reasons. Most seropositive subjects had LV systolic dysfunction considered mild (LVEF ranging between 40 and 49%, n = 17, 3.4%) or moderate (LVEF from 30–39%, n = 11, 2.2% of total); only 8 (1.6%) showed markedly depresses LVEF (<30%). Seropositive blood donors with and without LV dysfunction had comparable demographic and medical characteristics, although NT-proBNP levels were higher in those with LVEF below 50% (45 [25–76] vs351 [109–789], p<0.001).Seropositive donors with LV dysfunction showed longer PR, QRS and corrected QT intervals/durations (Figure 1), although both the heart rate and HRV indexes were not different between groups (data not shown). Both QRS and QTc duration were associated with LVEF values (rs: −0.159, p<0.0003, rs: −0.142, p: 0.002), and showed moderate accuracy in the detection of reduced LVEF (ROC AUC: 0.778 and 0.790, both p<0.0001, Figure 2). None of the other quantitative ECG variables showed significant correlation with measured LVEF.

Several ECG abnormalities were more commonly found in seropositive donors with depressed LVEF (table 3), including rhythm disorders (frequent supraventricular ectopic beats, atrial fibrillation or flutter and pacemaker), intraventricular blocks (right bundle branch block and left anterior fascicular block) and ischemic abnormalities (old myocardial infarction and major and minor ST abnormalities).

**Table 3:** ECG findings in *T.cruzi* seropositive blood donors by LVEF.

ECG variables	LVEF<0.50	LVEF> = 0.50	p-value*
	N = 36	N = 461	
**Rhythm**			
Sinus tachycardia	0	0	
Sinus bradycardia	1 (3)	20 (4)	
Frequent VPB	2 (6)	10 (2)	
Frequent SPB	3 (8)	6 (1)	0.02
Atrial fibrillation or flutter	2 (6)	0	0.005
Pacemaker	3 (8)	2 (<1)	0.003
**Intraventricular block**			
LBBB	1 (3)	0	
RBBB	10 (28)	70 (15)	0.04
LAFB	13 (38)	61 (13)	<0.0001
LAFB+RBBB	9 (25)	9 (2)	<0.0001
Incomplete LBBB	0	13 (3)	
Incomplete RBBB	5 (14)	30 (7)	
**Atrioventricular block**			
First degree	2 (6)	12 (3)	
Second degree	0	1 (<1)	
Third degree	0	0	
**Ischemic**			
Old MI	4 (11)	8 (2)	0.008
Major isolated ST-T abnormalities	7 (19)	16 (3)	<0.0001
Minor isolated ST-T abnormalities	12 (33)	47 (10)	<0.0001
ST segment elevation	0	20 (4)	
**Other**			
LVH	0	1 (<1)	
Short PR interval	0	16 (3)	
Major QT prolongation	1 (3)	2 (<1)	
Minor QT prolongation	1 (3)	7 (2)	
Low QRS amplitude	1 (3)	16 (3)	

* Reported if <0.05. VBP: ventricular premature beats; SPB: supraventricular premature beats; LBBB: left bundle branch block; RBBB: right bundle branch block; LAFB: left anterior fascicular block; Old MI: Old myocardial infarction, defined as major Q wave or minor Q waves with ST-T abnormalities; LVH: Left ventricular hypertrophy; major QT prolongation: QTi>115%; minor QT prolongation: QTi>111%; QTi: QT prolongation index: (QT/656) * (HR+100).

Almost all seropositive donors (33/36) with depressed LVEF showed at least one major or minor ECG abnormality. Only three subjects presented with normal ECG and abnormal LVEF: 0.46. 0.45 and 0.40, respectively; this last subject is a hypertensive patient with typical features of hypertensive cardiomyopathy in the Echo study who was not classified as having Chagas cardiomyopathy by the expert adjudication process employed in the parent study [19]. Seropositive donors with depressed LVEF had a higher prevalence of ECG abnormalities (69% vs. 23%, p<0.001) and a higher number of major ECG abnormalities per tracing when compared to seropositive donors with normal LVEF (39% vs. 19% with one and 31% vs. 4% with 2 or more abnormalities, p<0.001)

The diagnostic performance of selected ECG abnormalities was evaluated (table 4). An abnormal ECG (with minor or major abnormalities) is a sensitive marker for recognition of seropositive donors with depressed LVEF, with a negative predictive value of 99% (96–100%).

**Table 4:** Diagnostic performance of selected ECG findings in the recognition of reduced left ventricular ejection fraction (<0.50) in *T.cruzi* seropositive blood donors.

ECG variable	Sensitivity	Specificity	Positive predictive value	Negative predictive value
**Abnormal ECG**	92 (76–98)	45 (40–50)	12 (8–16)	99 (96–100)
**Wide QRS (> = 120 ms)**	47 (30–64)	83 (79–97)	18 (11–28)	95 (93–97)
**Prolonged QTc (>440 ms)**	56 (38–72)	79 (75–82)	17 (11–25)	96 (93–97)

Data is presented as median (interquartile range) * Reported if <0.05** HRV data available in 24 seropositive subjects with LVEF<0.50 and 433 with LVEF> = 0.50. seropositive and 458 seronegative subjects. QTc: corrected QT interval by Bazett's formula; HRV: heart rate variability; rmssd: root mean square of successive differences in normal RR intervals; sdnn: standard deviation of all normal RR intervals.

## Discussion

In this analysis of ECG findings from a large, controlled study focused on former blood donors with and without *T. cruzi* seropositivity, the frequency of major ECG abnormalities was higher in seropositive donors than in seronegative subjects; right bundle branch block and/or left anterior were more commonly found in seropositive than in well-matched seronegative donors. When considering only seropositive donors, those with LV dysfunction rarely presented with a normal ECG, having one or more major ECG abnormalities, including rhythm disturbances, intraventricular blocks and ischemic abnormalities. The presence of either major or minor ECG abnormalities is therefore a sensitive marker of the presence of LV dysfunction in ChD, and, equally important, the absence of ECG abnormalities has high negative predictive value. Consequently, we recommend that initial clinical evaluation of seropositive blood donors (and probably seropositive subjects identified through other population based screening programs) can be limited to ECG with more expensive echocardiography performed on patients with abnormal ECG and/or clinical findings suggesting of ChD.

Our findings suggest that seropositive blood donors have a similar profile to community ChD populations in terms of the prevalence and type of ECG abnormalities [12], [14], [15], [24], albeit with significantly lower rates and severity of abnormalities than in hospital and clinic-based series [11], [16], [16], [25]–[28]. However, the mean age of blood donors was higher than those studied in early community-based series and the prevalence of concurrent chronic conditions in blood donors was relatively high (24% hypertension, 5% diabetes), in contrast to some studies in which those conditions were excluded [16], [28]. Considering this higher frequency of ECG abnormalities in seronegative donors, right bundle branch block and/or left anterior fascicular block was the only finding more frequently found in seropositive subjects in relation to well-matched seronegative controls. This was also observed by Williams-Blandero et al. [14], in one of the most recent of those community-based studies, with a similar age profile. Indeed, since the interruption of vector-mediated transmission has been achieved in many Latin American countries [2], the age of *T. cruzi* infected subjects is increasing, and ChD is now a public health problem among older individuals in previously endemic regions [29].

LV systolic dysfunction, defined as reduced LVEF (<0.50), is a major marker of higher risk of death in ChD [30]. LV dysfunction, generally mild or moderate, occurred in a minority of cases in this sample (7.2%), reflecting the low risk profile of the seropositive donor population studied (the donors had to be clinically asymptomatic to give blood donations in 1998–2002, 8–12 years prior to the rigorous assessment including ECG and Echo that was the basis of the current analysis). In contrast, Ribeiro et al. [31] found a prevalence of more severe LV dysfunction (defined as LVEF≤0.40) of 9.1% and 14.5% in two samples from a Brazilian Outpatient Clinic that is a regional reference center for blood banks and primary care units, and Salles et al. [28] observed LV dysfunction in 109 out of 738 patients (14.8%) at another Brazilian Reference Outpatient Clinic.

In this study, several ECG abnormalities typical of ChD were predictive for LV dysfunction among seropositive donors. Right bundle branch block, frequently combined with left anterior fascicular block, is the most characteristic ECG abnormality in ChD [10] and is associated with higher risk of death in longitudinal studies [13], [32]. ChD patients with pacemakers have lower LVEF comparative to pacemaker patients without ChD [33]. In contrast to what we found in this study, frequent ventricular ectopic beats have been repeatedly related to LVEF depression in ChD [26], [34] and the observed association of supraventricular ectopic beats with LV dysfunction has not been reported before. Since frequent supraventricular ectopic beats can precede the development of atrial fibrillation, we hypothesize that higher left atrial pressure and volume secondary to LV systolic dysfunction may lead to frequent atrial ectopic beats and, after years, to atrial fibrillation. Atrial fibrillation is a late abnormality in the natural history of ChD, related to other ECG abnormalities, LV dysfunction and higher risk of death [12], [35]. Pathological q waves, ischemic ST-T abnormalities and abnormal T waves have also been reported to be markers of risk in Chagas cardiopathy [27], [36]. Prolonged QRS duration and QTc interval were both related to LV dysfunction [27], [37] and to worse prognosis [27], [38]. Since prolonged excitation time in ventricular conduction defects may induce secondary prolongation of the QT interval [39], the significance of prolonged QTc interval in seropositive donors should be interpreted with caution, considering that no correction for QRS duration was made in this study. Those with more than one ECG abnormality are at greater risk of having LV dysfunction, as reported in other studies [26], [40].

Heart rate variability indexes SDNN and RMSSD, calculated from standard 10-seconds ECG tracing, were reduced in seropositive donors but these findings were not correlated to the presence of LV systolic dysfunction. Both SDNN and RMSSD indexes from a 10-second ECG are markers of parasympathetic modulation of the sinus node [41]. In some studies in other settings [42]–[44], 10-s HRV indexes were predictive of the risk of death. Impairment of cardiac vagal modulation has been consistently reported in ChD [45]–[47] and occurs early in the evolution of the disease, preceding LV systolic dysfunction [48]. However, the association of vagal dysautonomia with LVEF and with prognosis in ChD is still controversial [46], [49].

Data from our study suggest that the ECG can be useful to guide the management of seropositive blood donors: an abnormal ECG is a sensitive marker of LV dysfunction, while a normal ECG carries a high negative predictive value. Indeed, a normal ECG is an established marker of excellent prognosis in medium-term follow-up of *T. cruzi* seropositive subjects [12], [13], [16]. The diagnostic performance of a single ECG interval measurement, such as a normal QRS duration or corrected QT interval, is not good, as previously reported [37].

The main strength of this analysis is that it is based on findings from a large controlled, rigorously conducted study, with central and blinded reading of both ECG and Echo results, and with classification using an internationally accepted ECG code, the Minnesota Code. This is, to the best of our knowledge, the only study with these features in a population of seropositive and matched seronegative blood donors. Because these blood donors were healthy at study baseline, the incidence of pathology will be lower than in prevalent patient cohorts. On the other hand, because patients with common chronic conditions, including hypertension and diabetes, were not excluded, it allows the generalization of findings to other seropositive blood donor populations in endemic and non-endemic countries. The main limitations are related to the cross-sectional status of the current analysis, with no information on the prognostic role of the observed abnormalities. We also lacked baseline measurements on the cohort to assure the absence of cardiac pathology, although all were healthy enough to donate blood. Moreover, blood donors from non-endemic countries may have different epidemiological profiles due to country of origin, socio-economic status and time since acquisition of infection prior to detection as seropositive donors, as well as possible differences in disease progression depending on the strain of *T. cruzi*.

In conclusion, we identified several ECG abnormalities that are predictive of LV dysfunction in ChD. Due to the study setting involving previously healthy seropositive donors who developed incident ChD, these study findings may be extrapolated to other low-risk populations. In particular, the results may guide the evaluations of patients with incidentally detected *T. cruzi* seropositivity from blood bank testing in endemic and increasingly in non-endemic countries, and from public health screening in endemic countries.
